# Characterization of pulmonary immune responses to hyperoxia by high-dimensional mass cytometry analyses

**DOI:** 10.1038/s41598-020-61489-y

**Published:** 2020-03-13

**Authors:** D. Hanidziar, Y. Nakahori, L. A. Cahill, D. Gallo, J. W. Keegan, J. P. Nguyen, L. E. Otterbein, J. A. Lederer, S. C. Robson

**Affiliations:** 1 Department of Anesthesia, Critical Care and Pain Medicine, Massachusetts General Hospital, Boston, MA 02114 USA; 2 Department of Surgery, Brigham and Women’s Hospital, Boston, MA 02115 USA; 3 Department of Surgery, Beth Israel Deaconess Medical Center, Boston, MA 02115 USA; 4 Departments of Anesthesia and Medicine, Beth Israel Deaconess Medical Center, Boston, MA 02115 USA

**Keywords:** Acute inflammation, Respiratory distress syndrome

## Abstract

Prolonged exposure to hyperoxia has deleterious effects on the lung, provoking both inflammation and alveolar injury. The elements of hyperoxic injury, which result in high rates of lethality in experimental models, are thought to include multicellular immune responses. To characterize these alterations in immune cell populations, we performed time-of-flight mass cytometry (CyTOF) analysis of CD45-expressing immune cells in whole lung parenchyma and the bronchoalveolar space of mice, exposed to 48 hours of hyperoxia together with normoxic controls. At the tested time point, hyperoxia exposure resulted in decreased abundance of immunoregulatory populations (regulatory B cells, myeloid regulatory cells) in lung parenchyma and markedly decreased proliferation rates of myeloid regulatory cells, monocytes and alveolar macrophages. Additionally, hyperoxia caused a shift in the phenotype of alveolar macrophages, increasing proportion of cells with elevated CD68, CD44, CD11c, PD-L1, and CD205 expression levels. These changes occurred in the absence of histologically evident alveolar damage and abundance of neutrophils in the parenchyma or alveolar space did not change at these time points. Collectively, these findings demonstrate that pulmonary response to hyperoxia involves marked changes in specific subsets of myeloid and lymphoid populations. These findings have important implications for therapeutic targeting in acute lung injury.

## Introduction

Administration of supplemental oxygen is likely the most common medical intervention in critical care. High concentrations of inspired oxygen (up to 100%) are often administered to patients during thoracic and cardiac surgery and to critically ill patients with acute respiratory distress syndrome (ARDS) in order to achieve adequate oxygenation. However, it is now recognized that hyperoxia may have deleterious consequences by promoting inflammation, acute lung injury and impairing anti-microbial immunity^1–10^. Recent data also suggest that hyperoxia increases mortality of mechanically ventilated patients and patients with ARDS^11,12^.

Pulmonary responses to hyperoxia likely involve multiple immune cell populations. Separate studies have reported that hyperoxia inhibits macrophage proliferation^13,14^, heightens the ratio of F4/80^low^CD206^low^ (proinflammatory, “M1”) to F4/80^high^CD206^high^ (anti-inflammatory, “M2”) alveolar macrophages^15^, and promotes influx of CD11b^high^CD11c^low^ cells (“inflammatory monocytes”) and CD11b^high^CD11c^high^ macrophages (“exudative macrophages”) into the bronchoalveolar space^16^. Increased infiltration of parenchyma by invariant NKT cells^17^ and late recruitment of neutrophils^18^ have also been described. However, an integrative analysis of changes across all immune populations of the lung has not yet been undertaken.

To address this gap and develop further potential therapeutic targets, we used time-of-flight mass cytometry (CyTOF)^19^ to deeply phenotype immune cells in lung parenchyma and bronchoalveolar space of mice breathing room air and mice exposed to 48 hours of hyperoxia. At this early experimental time point, the exudative injurious phase of hyperoxic lung injury has not yet developed^20,21^ and in keeping with this, there was no histologic evidence of alveolar damage.

The systems immunology approach utilizing CyTOF allowed us to describe early immune cell compositional changes in the lung during hyperoxia. Characteristics of immune subsets (abundance, proliferation, phenotype) were contrasted between normoxia and hyperoxia using appropriate systems biology algorithms (viSNE, CITRUS)^22–25^; as well as traditional biaxial manual gating approaches. The innovative high-dimensional analysis revealed heterogeneous *in vivo* reactions of alveolar macrophages, parenchymal B cells, myeloid regulatory cells and other subsets with a less definitive lineage specification. Collectively, our findings illustrate the complexity of early immune-modulatory effects of hyperoxia and potentially have therapeutic implications for specific cell targeting in acute lung injury.

## Results

### viSNE analysis delineates immune cell composition of the lungs during normoxia and hyperoxia

**Figure 1:**
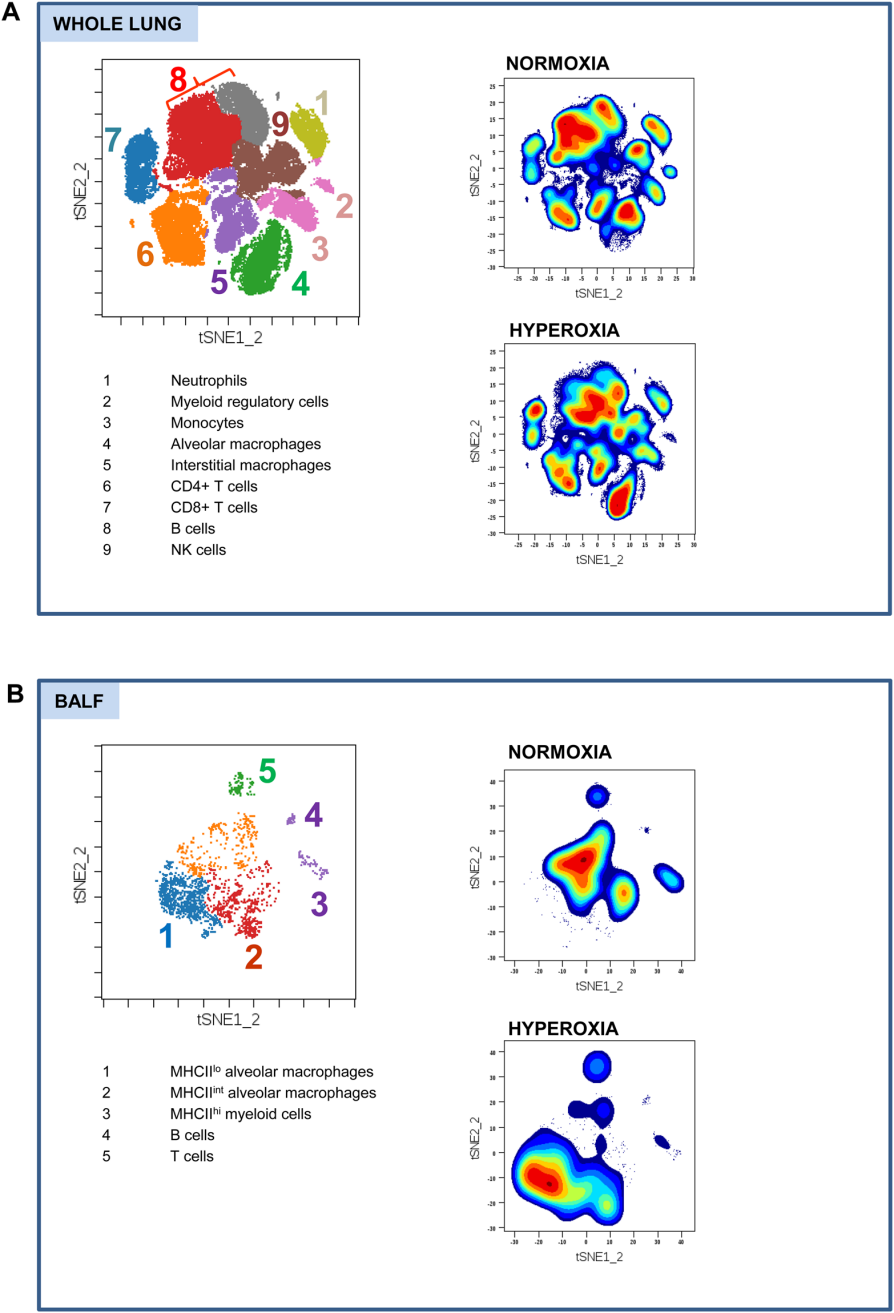
The viSNE analysis defines distinctive immune cell clusters in the lungs during normoxia and hyperoxia. (**A**) Whole lung major immune cell clusters^1–9^ during normoxia and hyperoxia. Presented tSNE plots were constructed based on data obtained from 7 mice in normoxia and 7 mice in hyperoxia. (**B**) BALF major immune cell clusters^1–5^ during normoxia and hyperoxia. Presented tSNE plots were constructed based on data obtained from 6 mice in normoxia and 8 mice in hyperoxia. FlowSOM was used to color-code the clusters. The immune lineages of the clusters are listed in the corresponding tables.

Immune cells were isolated from whole lung parenchyma, and in the independent experiment, from bronchoalveolar lavage fluid (BALF) of C57BL/6 mice breathing room air (FiO_2_ = 21%) and mice exposed to 48 hours of hyperoxia (FiO_2_ > 95%). At this early experimental time-point, there was no histologic evidence of lung parenchymal damage **(**Fig. S1**)**. Single-cell suspensions were stained with a panel of 36 metal isotope-tagged monoclonal antibodies **(**Table S1**)**. Multi-dimensional data were acquired by time-of-flight mass cytometry and analyzed with viSNE (visualization of t-distributed stochastic neighbor embedding) algorithm. Live CD45+ immune cells for analysis were identified by a sequence of gating steps **(**Fig. S2**)**. ViSNE analysis was run on equal numbers of events per each sample and data were pooled to generate final 2-dimensional density plots **(**Fig. 1**)**. In tSNE plots, immune cells are clustered based on their phenotypic similarity. This unbiased analysis identified 9 major clusters in lung parenchyma **(**Fig. 1A; clusters 1–5 representing myeloid cells and 6–9 lymphoid cells**)** and 5 major clusters in the bronchoalveolar space **(**Fig. 1B; clusters 1–3 representing myeloid cells and 4–5 lymphoid cells**)**. The immune lineage of clusters was determined based on their distinct expression pattern of surface and intracellular markers, further validated by overlays of manually gated populations on tSNE plots **(**Figs. S3 and S4**)**. The same numbers of major immune clusters were identified in lungs and BALF during normoxia and hyperoxia. BALF data revealed that there was no infiltration of neutrophils into the alveolar space at this early time-point. However, we note that distribution of several density clusters (e.g., alveolar macrophages, B cells) in relation to tSNE1 and tSNE2 axis was shifted during hyperoxia, indicating multicellular immune responses. We next sought to further characterize specific changes in these immune cell clusters induced by hyperoxia.

### Lung response to hyperoxia involves specific lymphoid and myeloid subsets

**Figure 2:**
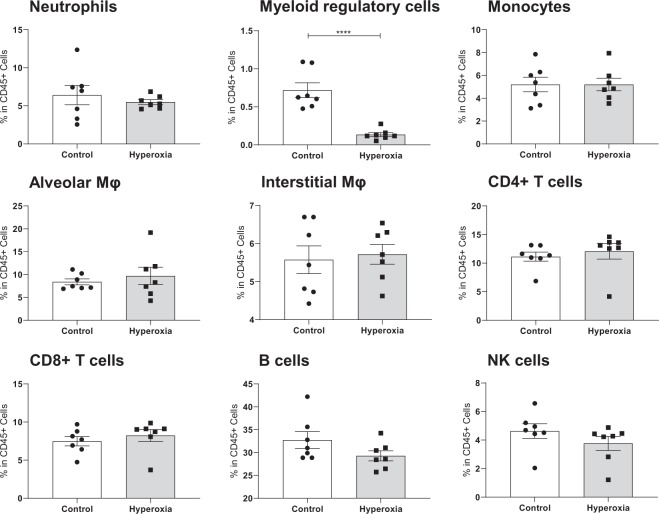
Proportions of major immune subsets in normoxia and hyperoxia. Frequencies of major subsets in whole lung during normoxia and hyperoxia, compared by unpaired t-test. Each dot represents data from one lung. ****Indicates p < 0.0001. Myeloid regulatory cells are found to be significantly reduced in hyperoxia.

We first compared frequencies (% CD45+ cells) of major immune cell subsets as defined by manual gating strategy between normoxia and hyperoxia. The absolute cell numbers within immune populations are reported in Fig. S6. This approach identified significant reduction of a distinct immune cell population with a myeloid regulatory cell phenotype (CD172a + CD11b^hi^ CD11c^lo^ Ly6C + Ly6G − Siglec-F + F4/80 + CD39+) in lung parenchyma **(**Fig. 2**)**, a change further evident in tSNE plots from two independent experiments **(**Fig. S5**)**.

**Figure 3:**
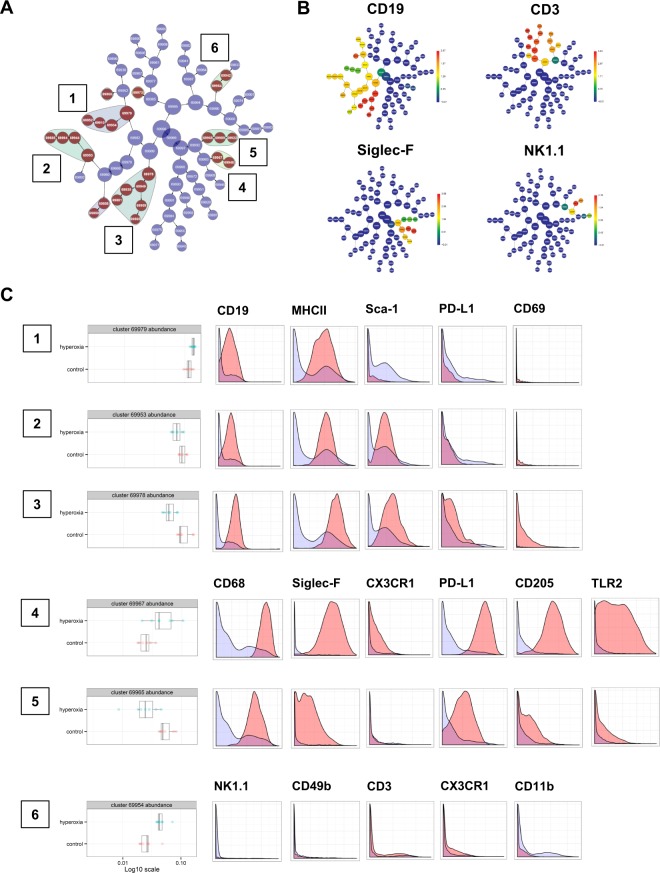
Unbiased identification of immune clusters modified by hyperoxia. (**A**) CITRUS algorithm identifies 6 major immune cell clusters^1–6^ where the abundance is significantly altered between hyperoxia and normoxia. (**B**) Expression of CD19, Siglec-F, CD3 and NK1.1 relevant for the clusters identified by CITRUS. (**C**) The boxplots indicate the spread of the abundance of the clusters, and the histograms depict the expression of specific cellular markers (blue depicts background expression in all cells, and red indicates marker expression of cells in a cluster).

To further explore whether abundance of other smaller subpopulations (not primarily identified by biaxial manual gating strategy) differs between normoxia and hyperoxia, we utilized an unbiased CITRUS (cluster identification, characterization, and regression) analysis of whole lung CD45+ cells. Significance analysis of microarrays (SAM) association model was selected. When applying the most stringent statistical cutoff of 0.01 FDR (false discovery rate), CITRUS analysis identified 6 clusters of interest which distinguished normoxic and hyperoxic lungs **(**Fig. 3A**)**. We next determined the phenotypic identity of these 6 clusters identified by CITRUS **(**Fig. 3B,C**)**.

Clusters 1–3 were interpreted as B cells by their expression of CD19 and MHCII. Cluster 3 additionally expressed higher levels of PD-L1 and CD69 consistent with regulatory B cell phenotype. Cluster 1 (Sca-1^lo^) was increased, while clusters 2 and 3 (both Sca-1^hi^) were decreased in hyperoxia (cluster 3 most significantly). Significant reduction of regulatory B cells was further evident in tSNE plots from two independent experiments **(**Fig. S7**)**. Clusters 4 and 5 were interpreted as alveolar macrophages by their high expression of CD68 and Siglec-F. Cluster 4, which was significantly increased in hyperoxia, expressed higher levels of CX3CR1, PD-L1, CD205 and TLR2 as compared to cluster 5. The abundance of cluster 5 alveolar macrophages was decreased in hyperoxia. Cluster 6, which was significantly increased, did not exhibit definitive lineage specification (low expression of CD3, NK1.1, CD49b and CD11b). Significant differences in the frequencies (% CD45+ cells) of the 6 clusters during normoxia and hyperoxia were additionally confirmed using unpaired t-test **(**Fig. S8**)**.

**Figure 4:**
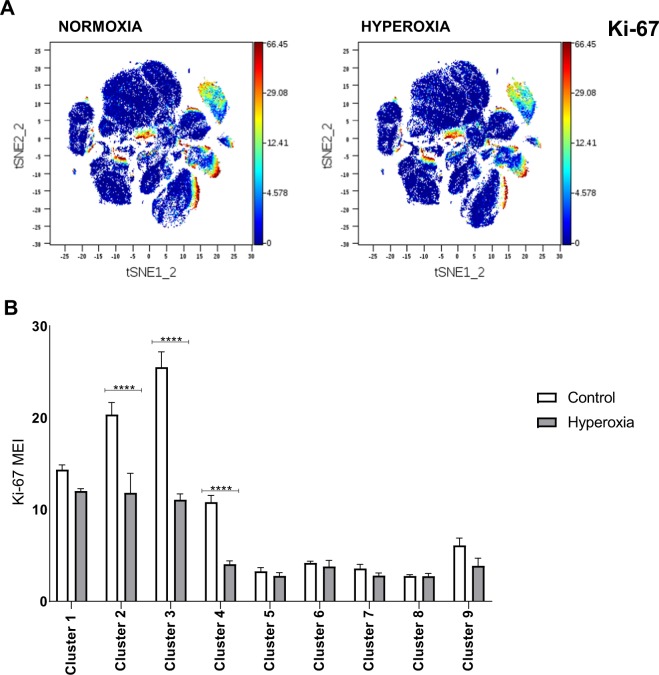
Proliferation of immune cell subsets in normoxia and hyperoxia. **(A)** ki67 staining in tSNE plots from normoxia and hyperoxia. **(B)** MEI of ki67 from equal sampled fcs files were exported for all 9 major clusters and compared using two-way ANOVA. Proliferation rate of myeloid regulatory cells (cluster 2), monocytes (cluster 3) and alveolar macrophages (cluster 4) is significantly reduced in hyperoxia. ****Indicates p < 0.0001.

We next analyzed ki67 protein expression to compare proliferation rate of major nine immune cell clusters in the lungs during normoxia and hyperoxia. We found a significantly reduced proliferation rate of myeloid regulatory cells, monocytes and alveolar macrophages during hyperoxia, while proliferation rates of other populations did not change significantly **(**Fig. 4**)**.

### Hyperoxia promotes an aberrant macrophage phenotype in the alveolar space

**Figure 5:**
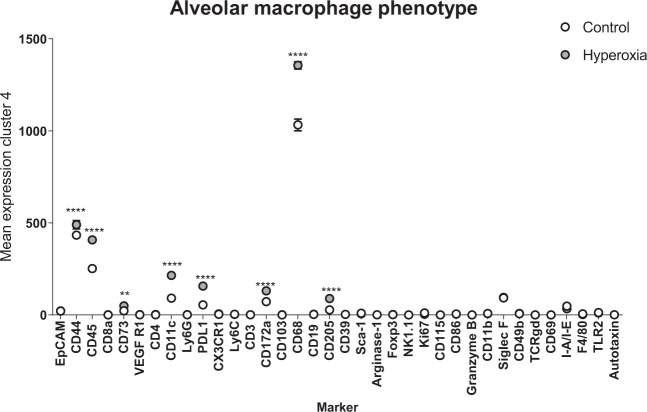
Molecular profile of a bulk alveolar macrophage population in normoxic and hyperoxic lung. Comparison of mean expression levels of select markers by alveolar macrophages during normoxia and hyperoxia using two-way ANOVA. **Indicate p < 0.01, ****Indicate p < 0.0001.

**Figure 6:**
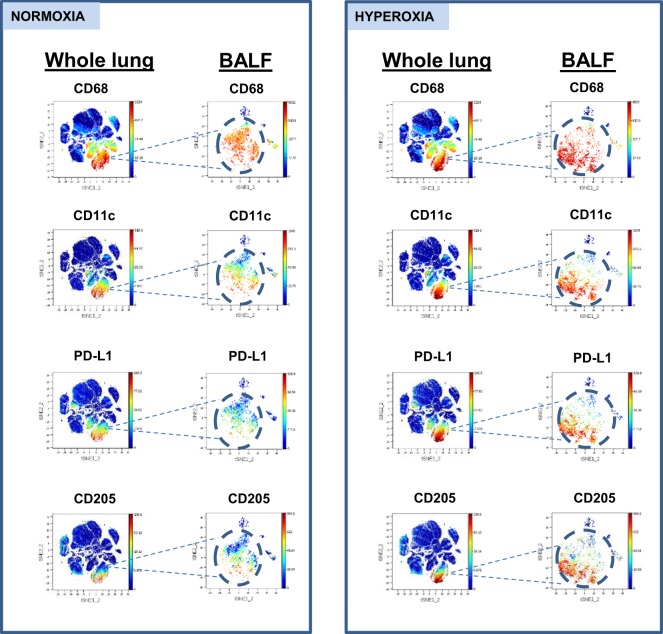
Single cell expression of alveolar macrophage markers in normoxic and hyperoxic lung. Parallel tSNE plots depict expression of select macrophage markers in whole lung and BALF during normoxia and hyperoxia. tSNE plots from whole lung and BALF are obtained from independent experiments.

Given the complex changes involving populations of alveolar macrophages, we next investigated their phenotype in greater detail. Macrophages were the dominant population in the alveolar space during normoxia and after 48 hours of hyperoxia. We compared expression levels of select markers on the entire pool of alveolar macrophages in normoxia and hyperoxia. We found significant up-regulation of CD44, CD45, CD11c, CD68, CD172a, CD73, CD205, PD-L1 in hyperoxia, consistent with an activated macrophage phenotype **(**Fig. 5**)**. Increased expression of these markers at a single cell level during hyperoxia was also confirmed by tSNE plots of CD45+ cells from whole parenchyma and CD45+ cells from BALF, obtained from 2 separate experiments **(**Fig. 6**)**. Collectively, our data indicate that the aberrant macrophages expressing elevated levels of PD-L1, CD68, CD11c and CD205, infrequent in normal alveoli, become enriched during hyperoxia.

Small populations of myeloid cells (MHCII^hi^), T cells (CD3+) and B cells (CD19+) were present in the alveolar space during both normoxia and hyperoxia. Phenotypic changes of these small intra-alveolar populations were not detected in our study.

## Discussion

High inspired concentrations of oxygen are frequently administered to anesthetized or critically ill patients to ensure adequate tissue oxygenation. However, hyperoxia may promote inflammation, contribute to acute lung injury and impair antimicrobial immunity. Hyperoxia is thought to trigger a multicellular immune response, implicating monocytes, macrophages, neutrophils and NKT cells, as noted in separate studies^13–18^. However, a global view of the immune populations during hyperoxia has not been previously studied.

The key contribution of our study is an unbiased identification of immune populations which respond to hyperoxia by changes in their abundance, proliferation rate or phenotype. With an intent to identify the early responses to hyperoxia, the immune populations were analyzed at 48 hours of hyperoxia exposure, before the exudative phase of acute lung injury is developed. No evidence of parenchymal damage was found histologically at this experimental time point. In the mouse model of hyperoxia used by us and other authors, lung injury becomes evident histologically at 72 hours, with further hyperoxia exposure leading to uniformly lethal lung injury at 80–90 hours^17,20^.

By applying CyTOF mass cytometry approach, we described previously unrecognized heterogeneous effects of hyperoxia, including, 1) reduction of immune regulatory populations (parenchymal regulatory B cells and myeloid regulatory cells), 2) diminished proliferation rate of myeloid regulatory cells, monocytes and alveolar macrophages, and 3) enrichment of macrophages with an aberrant phenotype in the alveolar space. At this tested time point, no increases in neutrophil population were observed.

In our study, myeloid regulatory cells have been identified as a distinct cluster of CD172a + Siglec-F + CD68 − F4/80 + CD11b^hi^ CD11c^lo^ Ly6C + Ly6G − CD39+ cells. The lack of CD68 expression differentiated these innate immune cells from pulmonary monocytes and macrophage subsets, and lack of Ly6G differentiated them from neutrophils. The expression of Siglec-F and F4/80 on some of these cells indicates close phenotypic similarity with, and/or admixture, of eosinophilic granulocytes^26–29^. Expression of CD39 (ENTPD1), an ectoenzyme hydrolyzing extracellular adenosine triphosphate, supports immunoregulatory activity of this population^30,31^. Our observation that hyperoxia adversely affects the size of this population mirrors observations in tumor immunology. Hyperoxia has anti-tumor effects, at least in part, by depleting myeloid derived suppressor cells (MDSC) from tumors^32^. In contrast, hypoxia promotes maintenance of MDSC in tumor tissues^33^. From these and our studies, we might conclude that myeloid regulatory cells are highly sensitive to oxygen tension *in vivo*. Whether depletion of myeloid regulatory cells promotes lung inflammation, or is merely a bystander effect, requires further studies.

Regulatory B cells were identified as a distinct cluster of CD19+ B cells expressing high levels of PD-L1. Regulatory B cells are a subset of B cells exerting immunoregulatory suppressive functions, in part, by engaging PD-1 on target cells^34,35^. Further characteristics included high levels of CD69 and Sca-1, consistent with an activated/memory phenotype^36,37^. We speculate that the early depletion of pulmonary immunoregulatory populations such as regulatory B cells and myeloid regulatory cells may facilitate development of tissue-destructive inflammation (e.g., NKT cell, neutrophil infiltration) during later stages of hyperoxic lung injury. Our data infer association only but do provide new avenues of study.

Hyperoxia was previously found to inhibit macrophage proliferation *in vitro*^13^ and *in vivo*^14^. We therefore analyzed ki67 protein expression in all major immune cell clusters. We have found a significantly diminished proliferation rate of alveolar macrophages, myeloid regulatory cells and monocytes during hyperoxia. Hyperoxia did not alter proliferation of other immune populations at the tested time point.

Contributions of alveolar macrophages to the evolution of hyperoxic lung injury are not fully understood. We found that hyperoxia promotes an aberrant macrophage phenotype with elevated expression of multiple markers, including CD44, CD68, CD11c, CD73, PD-L1 and CD205. The functional consequences of this phenotypic shift have not been investigated in the present study. However, we speculate that the upregulation of PD-L1 by macrophages may be an early adaptive response to counteract oxygen-induced alveolar inflammation. PD-L1 delivers an inhibitory signal to macrophages and other immune cells^38^, perhaps contributing to reduced proliferative rate of macrophages observed during hyperoxia by several authors^13,14^. In human ARDS, low expression of PD-L1 by alveolar macrophages is associated with prolonged mechanical ventilation and increased mortality^39^, indicating importance of PD-1/PD-L1 pathway in regulating the evolution of lung injury.

Importantly, we cannot exclude that other populations of lung immune cells undergo significant changes during hyperoxia, however, these changes were not captured by our analytic approach. This may be due to the limitations of CyTOF, our chosen panel of antibodies, or a chosen time-point. For instance, we found that proportion of neutrophils remains unchanged at 48 hours of hyperoxia and neutrophils did not migrate into the alveolar space. This is in contrast with significant expansion of neutrophils that we previously described in later stages of hyperoxic lung injury, at 72 hours of hyperoxic exposure^17^. The late infiltration by neutrophils coinciding with development of lung injury has also been shown by other authors^18^. In the present study, we did not identify a distinct cluster of NKT cells, likely due to small numbers of these cells in normal lungs as well as at 48-hour time point. Similar to neutrophils, we have previously reported significant NKT cell expansion at 72 hours of hyperoxia^17^.

No targeted therapies currently exist to inhibit lung inflammation and injury due to oxidative stress. Clinical management of patients with lung failure due to ARDS is currently only supportive and often involves administration of supraphysiologic concentrations of oxygen. It is therefore crucial that immune pathways linking hyperoxia, inflammation and organ injury are better characterized to allow and hasten development of therapies.

Our findings highlight the need to consider a variety of potential immune cell targets (macrophages, B cells, myeloid regulatory cells) when designing and testing immune-targeted therapies for acute lung injury or ARDS. Unbiased, systems immunology approaches like the ones used in this study may accelerate the discovery and development of immune-modulating therapies for sepsis and ARDS^40–42^.

## Methods

### Animals and exposure to hyperoxia

Eight-week-old male wild-type C57BL/6 mice were purchased from Jackson laboratory (Bar Harbor, ME) and were housed in accordance with guidelines from the American Association for Laboratory Animal Care. Mice between ages 9 and 16 weeks were used for experiments. For hyperoxia exposure, mice were placed in cages in a customized disinfected Plexiglas chamber, and continuous oxygen exposure was performed for 48 hours as previously published by our group^17^. A calibrated oximeter was placed in the chamber for a continuous measurement of FiO_2_. The inspired fraction of oxygen was maintained at above 95% by a fresh flow of oxygen at 2–3 L/min. Mice had unlimited access to food and water and the typical light-dark cycle was maintained. The hyperoxia exposure took place in a barrier animal facility. Our previous studies have determined that this exposure to hyperoxia results in severe lung injury and respiratory distress that would require euthanasia at 70–90 hours of exposure. The experiments received approval from the Beth Israel Deaconess Medical Center IACUC. All procedures involving animals were conducted in accordance to US federal guidelines in the AAALAC accredited animal facility.

### Histology

Whole lungs were harvested, fixed in neutral buffered formalin and embedded in paraffin. Tissue sections were stained with hematoxylin and eosin (H&E) using standard protocol^43^.

### Isolation of immune cells from whole lung and bronchoalveolar fluid

To obtain immune cells from whole lung, mice were deeply anesthetized by intraperitoneal injection of ketamine and xylazine^44^ and lungs were excised through thoracotomy approach. Upon excision, pulmonary vasculature was not perfused in order to allow inclusion of potential intravascular immune cells in subsequent analysis. Mediastinal lymph nodes and central airways were separated from the lungs because the goal was to analyze pulmonary parenchyma. All lobes of the right lung were then subjected to gentle tissue digestion at room temperature for 10 minutes with Liberase TL enzyme (Roche) and mechanically dissociated through a 40-micron sieve to yield a single-cell suspension (~10^6^ cells). Cells were cryopreserved in Cryostor CS10 (BioLife Solutions, Bothell, WA) following the manufacturer’s protocol to be used for subsequent staining with CyTOF-ready antibodies^23^. Bronchoalveolar fluid was obtained from mice, which were first euthanized by CO_2_ inhalation. Lack of respiratory and cardiac activity was confirmed and 22 G i.v. cannula was inserted into trachea via neck incision. Lungs were lavaged three times with 1 mL of phosphate buffered saline, fluid collected, and cell suspension obtained, as previously described^45^. Cells were cryopreserved as described above for subsequent CyTOF antibody staining.

### CyTOF staining method

Cryopreserved cells were thawed at 37 °C, washed by centrifugation, plated, and immediately stained. All CyTOF stains were performed at room temperature in 96-well round-bottom polypropylene plates (Corning). After washing cells by centrifugation at 200 × g for 5 minutes, 5 μM cisplatin viability staining reagent (Fluidigm, South San Francisco, CA) was added for 5 minutes. After centrifugation, TruStain FcX Fc receptor blocking reagent (BioLegend) was added for 10 minutes. CyTOF antibodies were labeled using purified antibodies from several different suppliers (BioLegend [San Diego, CA], eBioscience [Waltham, MA], and R&D Systems [Minneapolis, MN])^23^. The CyTOF staining panels used in this study were custom labeled using MaxPar labeling kits (Fluidigm). The list of antibodies is shown in Table S1. The antibodies for cell surface staining were incubated with cells for 30 minutes. After fixation and permeabilization of cells using the FoxP3 Staining Buffer Set (eBioscience), cells were barcoded using palladium barcoding reagents and combined into a single sample for pooled intracellular antibody staining for 30 minutes^23,46^. After intracellular staining, the cells were fixed with 1.6% formaldehyde. To stain DNA, 18.75 μM iridium intercalator solution (Fluidigm) was added to the cells. Cells were subsequently washed and reconstituted in Milli-Q filtered distilled water with EQ four element calibration beads (Fluidigm) and then analyzed on a Helios CyTOF Mass Cytometer (Fluidigm)^23^.

### CyTOF analysis and statistics

CyTOF data were first normalized and then debarcoded using the open-source Normalizer and Debarcoding software. Data analysis was performed using Cytobank (Mountain View, CA) and the available analysis algorithms (viSNE, CITRUS). ViSNE is a dimensionality reduction algorithm allowing visualization of high-dimensional single cell data mapped onto two dimensions as a tSNE plot^22^. tSNE plots were utilized to visualize all components of lung immune system. To identify differences in the abundance of cellular subsets between control and hyperoxia in an unbiased manner, we used CITRUS software. CITRUS (cluster identification, characterization, and regression) is an algorithm designed for the fully automated discovery of statistically significant stratifying biological signatures within single cell datasets containing numerous samples across multiple known endpoints (e.g. normoxia vs hyperoxia)^25^. The CITRUS software performed the statistical analysis of the high-dimensional CyTOF data to identify significant clusters. For descriptive statistics (comparison of cell cluster frequencies), unpaired t-test was performed using Graphpad Prism 8.0.0. (La Jolla, California). P-values <0.05 were considered statistically significant.

## Supplementary information

Supplementary information

## Data Availability

All data generated or analyzed during this study are included in this published article (and its Supplementary Information files).
