# Supplementary material for: Characterization of pulmonary immune responses to hyperoxia by high-dimensional mass cytometry analyses
Source: Sci Rep. 2020 Mar 13;10:4677. doi: 10.1038/s41598-020-61489-y (PMC7070092; doi:10.1038/s41598-020-61489-y)
Supplement: Supplementary file 1 — Supplementary information [file 41598_2020_61489_MOESM1_ESM.pdf]

Characterization of pulmonary immune responses to hyperoxia by high-dimensional mass cytometry analyses

Hanidziar D,[1,5]* Nakahori Y,[2,5] Cahill LA,[2] Gallo D,[3] Keegan J,[2] Nguyen JP,[2] Otterbein LE,[3] Lederer JA,[2,6] & Robson SC[4,6]

[1]Department of Anesthesia, Critical Care and Pain Medicine, Massachusetts General Hospital, Boston, MA 02114

[2]Department of Surgery, Brigham and Women's Hospital, Boston, MA 02115

[3]Department of Surgery, Beth Israel Deaconess Medical Center, Boston, MA 02115

[4]Departments of Anesthesia and Medicine, Beth Israel Deaconess Medical Center, Boston, MA 02115

[5]these authors contributed equally

[6]senior authors

Figure S1

NORMOXIA

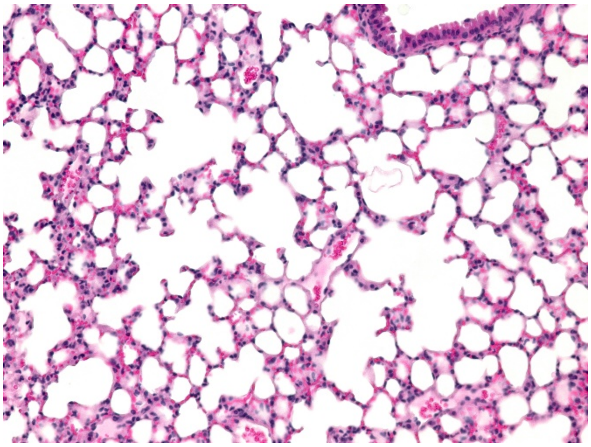

HYPEROXIA

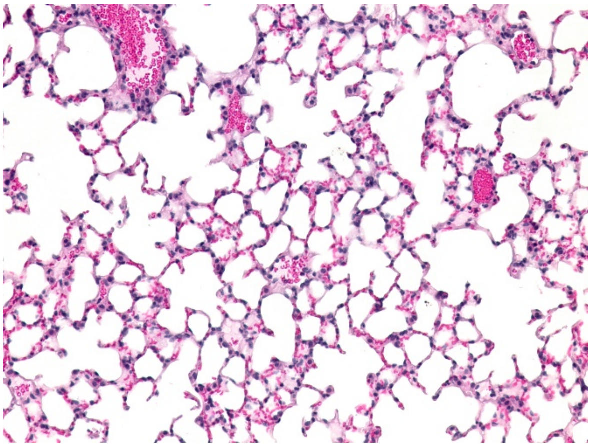

Figure S2

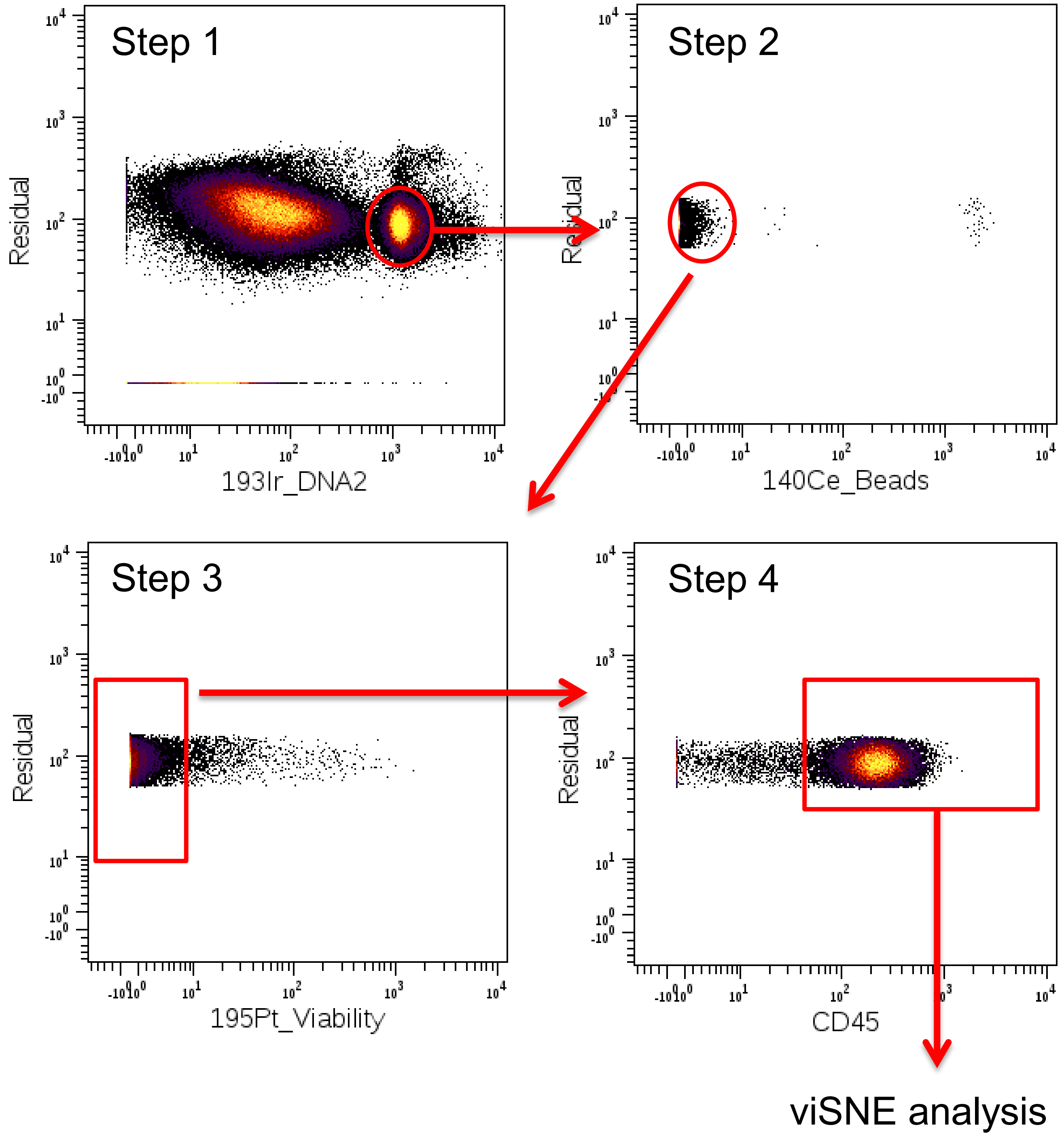

Step 1
Residual
193Ir_DNA2
Step 2
Residual
140Ce_Beads
Step 3
Residual
195Pt_Viability
Step 4
Residual
CD45
viSNE analysis

Figure S3

Cluster 1 Neutrophil

Manual gating

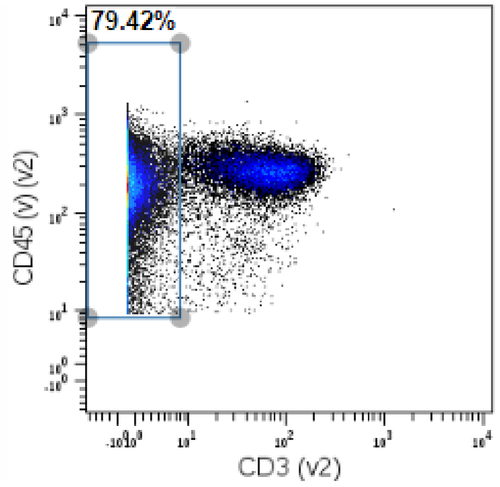

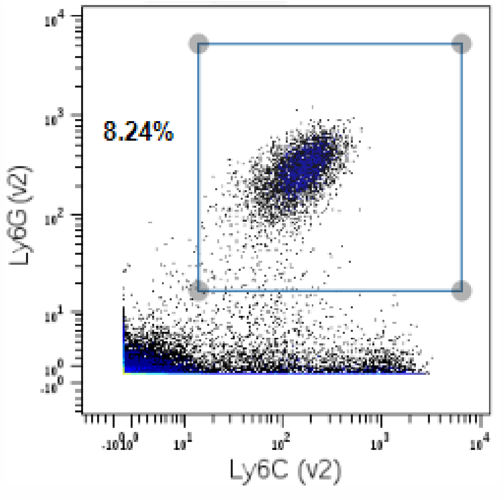

Overlay on viSNE

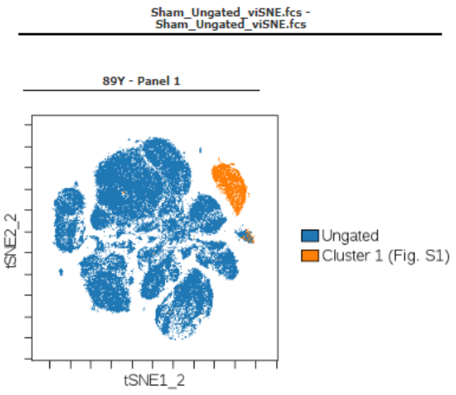

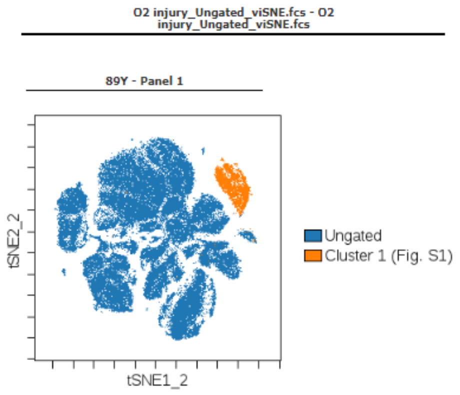

Expression of lineage markers

Ly6C

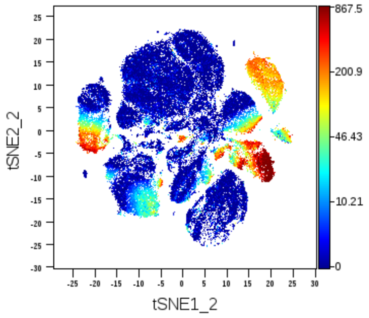

Ly6G

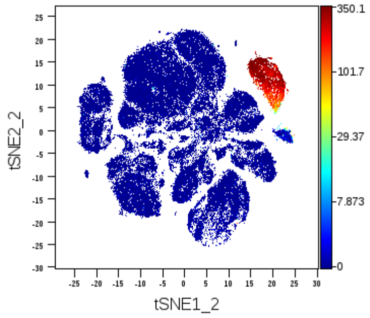

Cluster 2
Myeloid regulatory cell

Manual gating

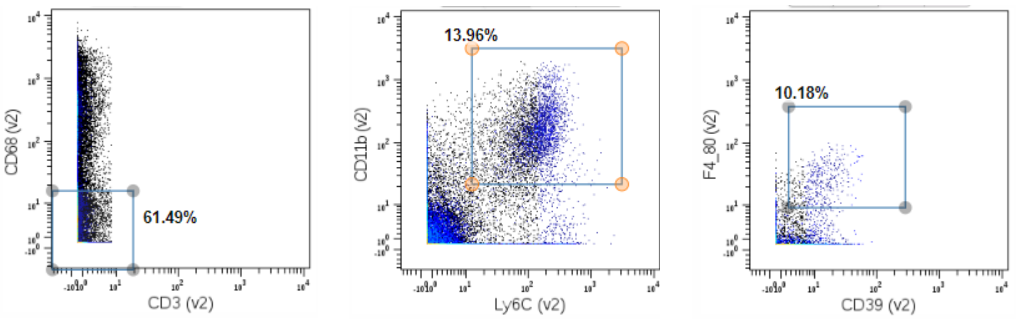

Overlay on viSNE

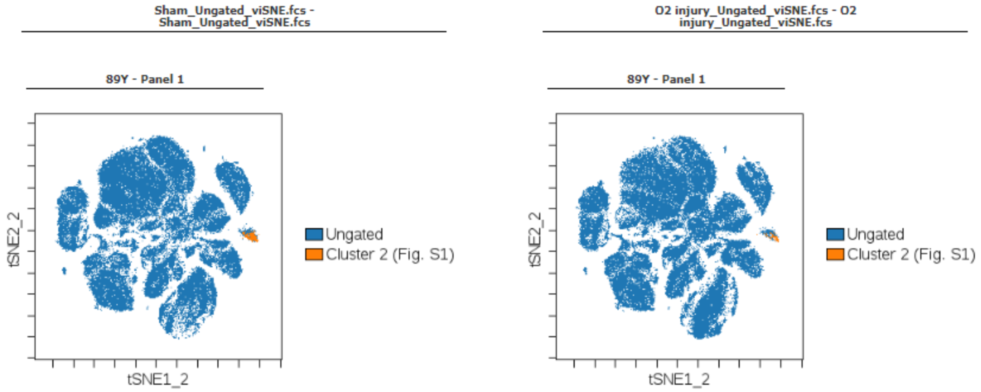

Expression of lineage markers

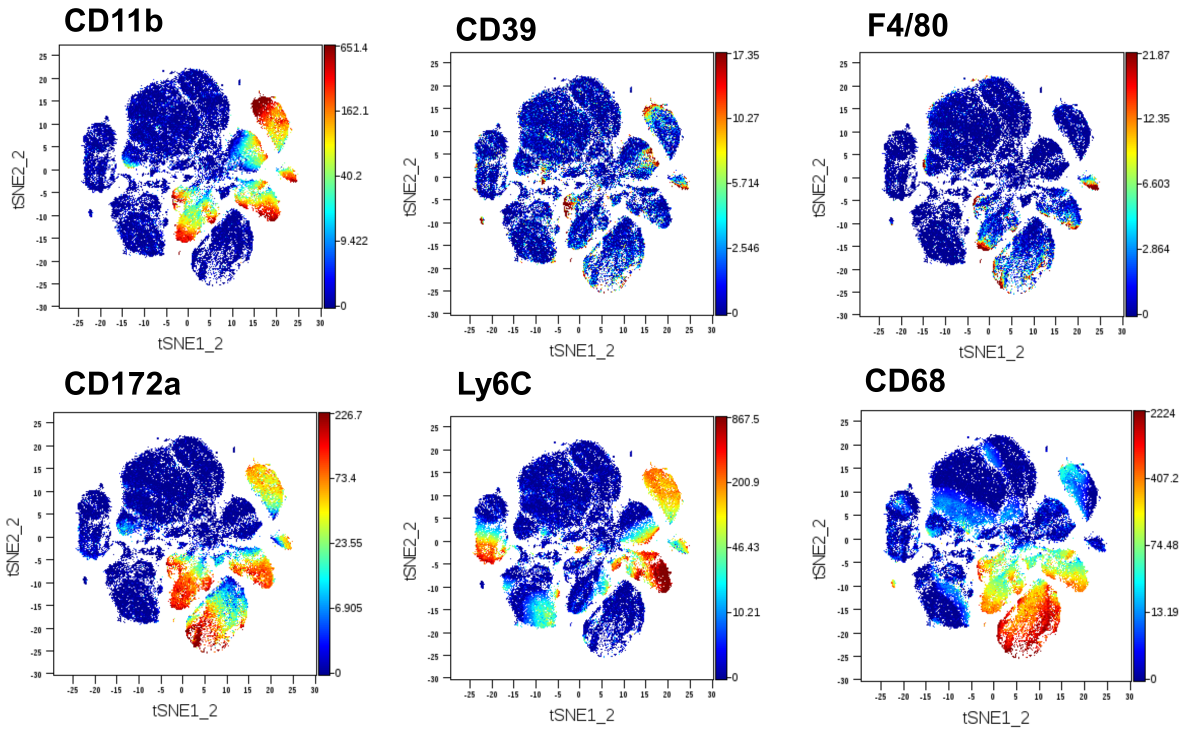

Cluster 3 Monocyte

Manual gating

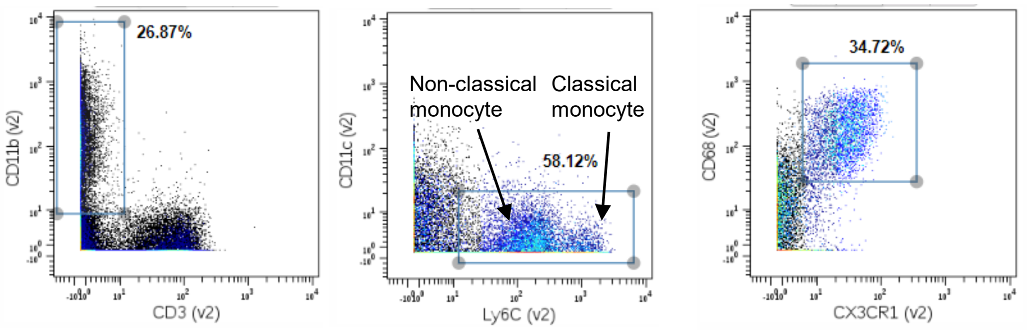

Overlay on viSNE

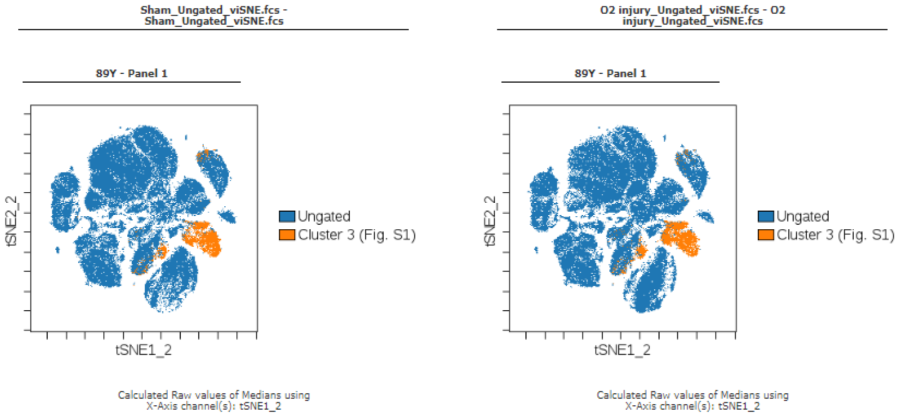

Expression of lineage markers

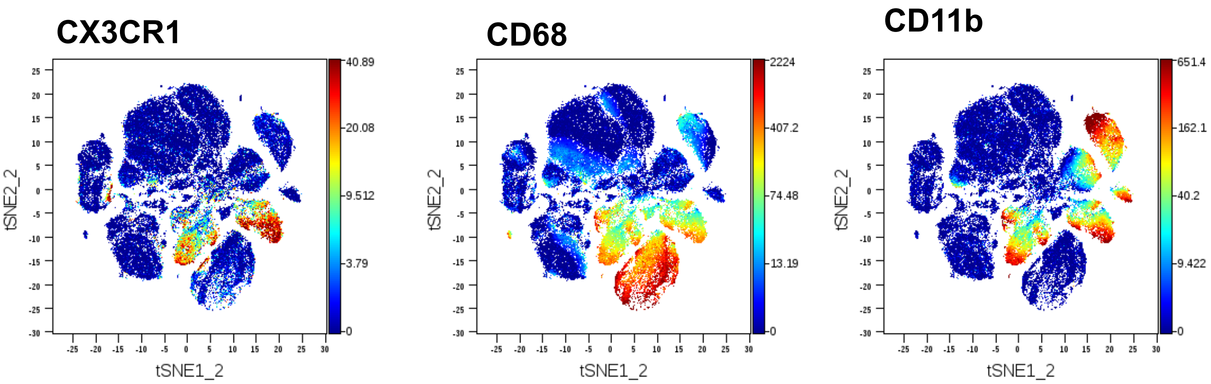

Cluster 4
Alveolar macrophage

Manual gating

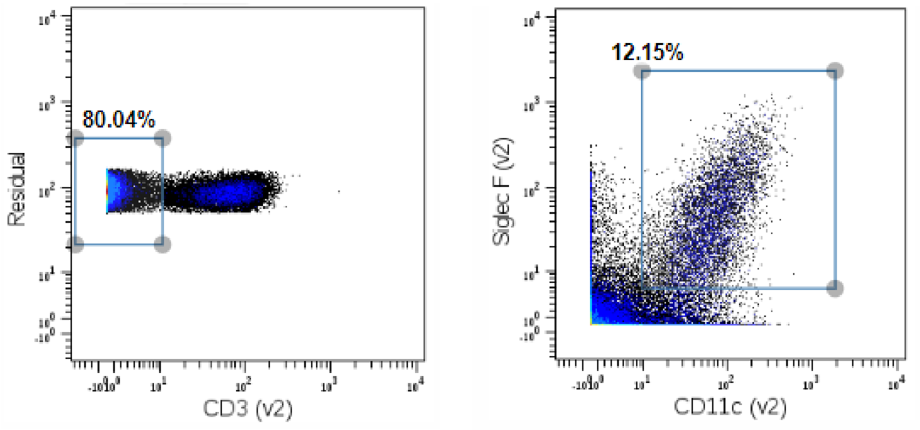

Overlay on viSNE

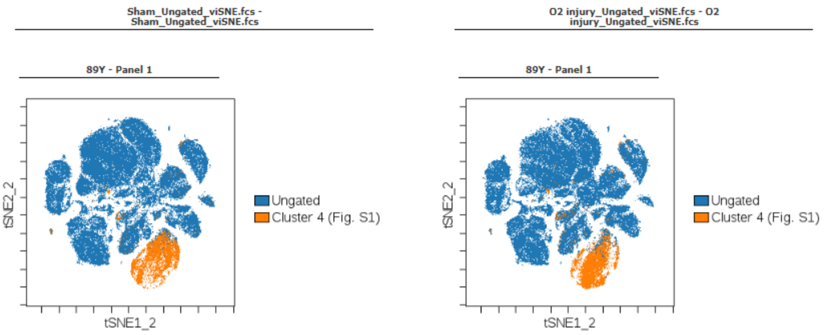

Expression of lineage markers

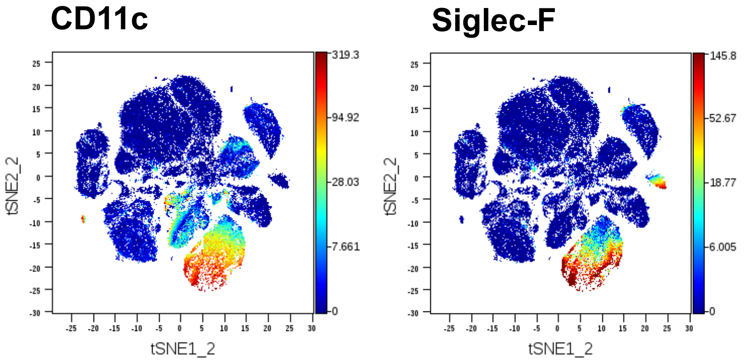

Cluster 5
Interstitial macrophage

Manual gating

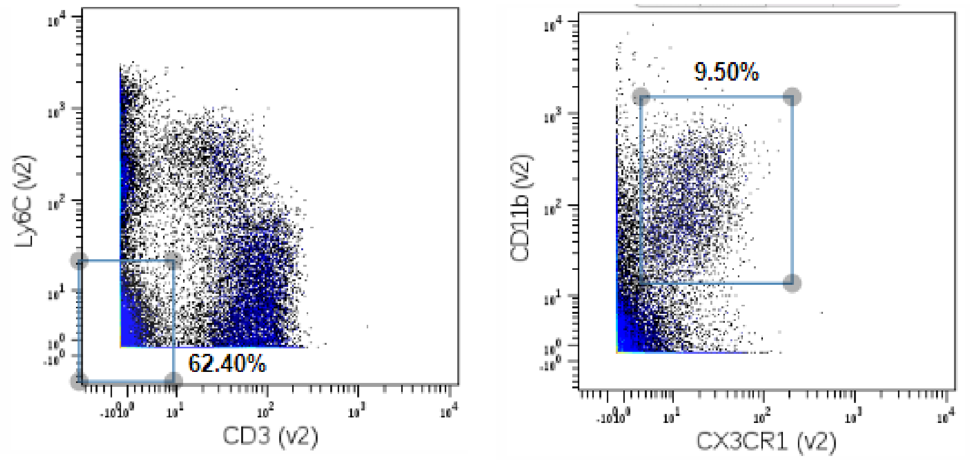

Overlay on viSNE

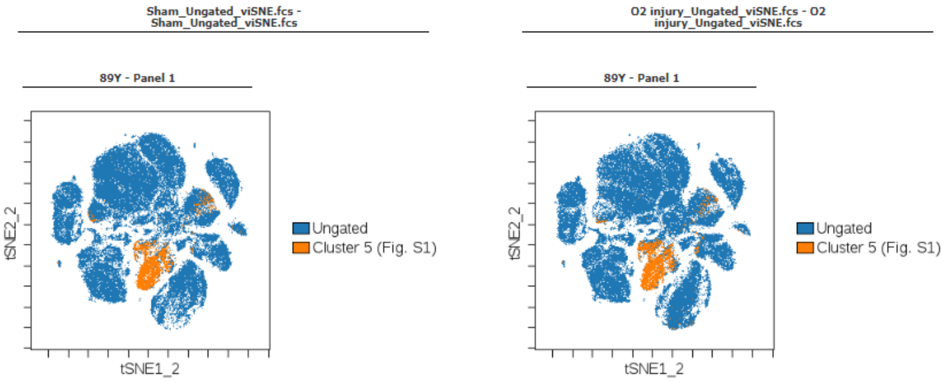

Expression of lineage markers

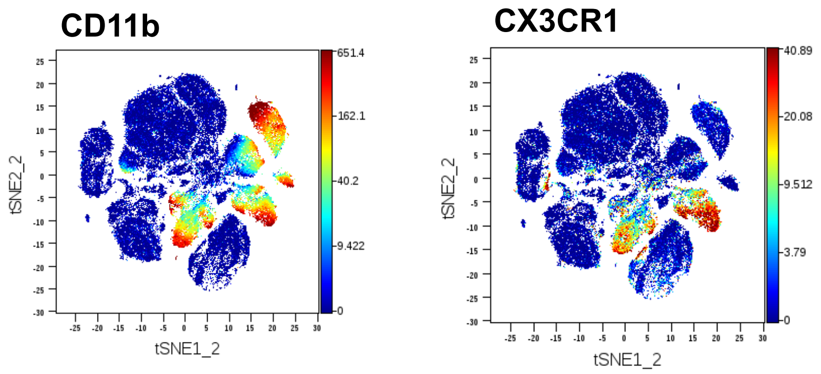

Cluster 6
CD4+ T cell

Manual gating

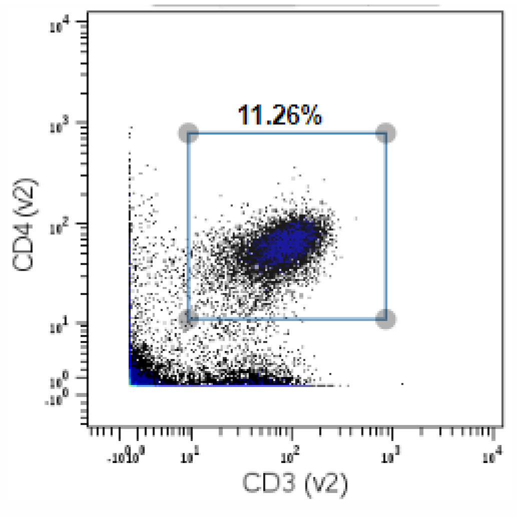

Overlay on viSNE

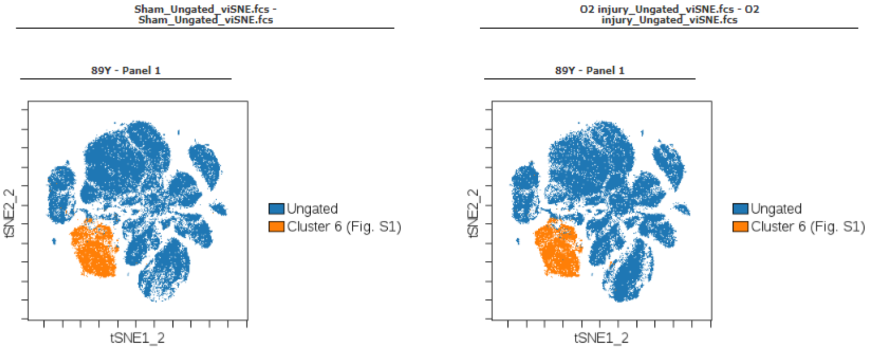

Expression of lineage markers

CD3 **CD4**

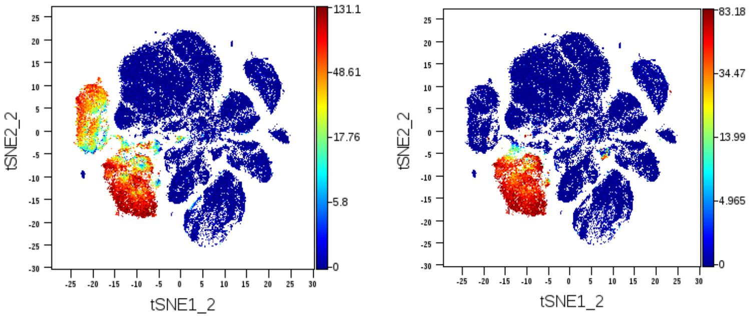

Cluster 7
CD8+ T cell

Manual gating

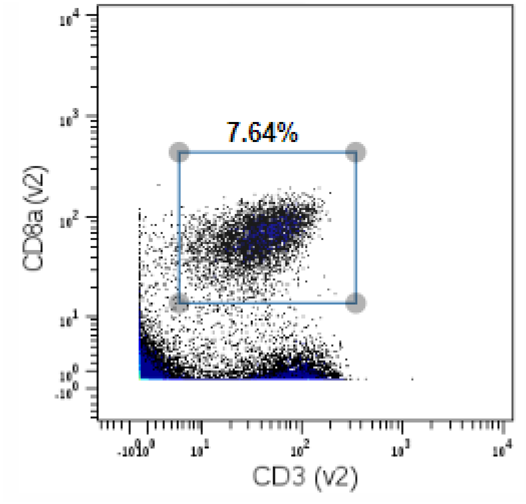

Overlay on viSNE

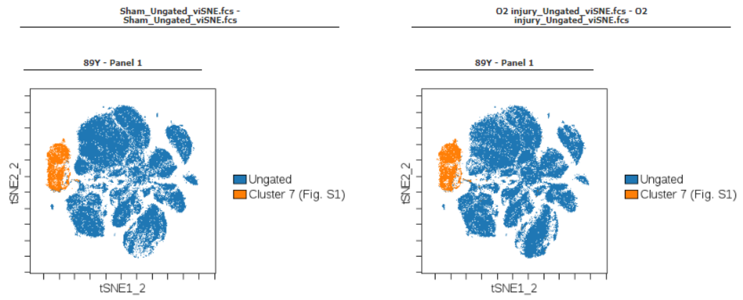

Expression of lineage markers

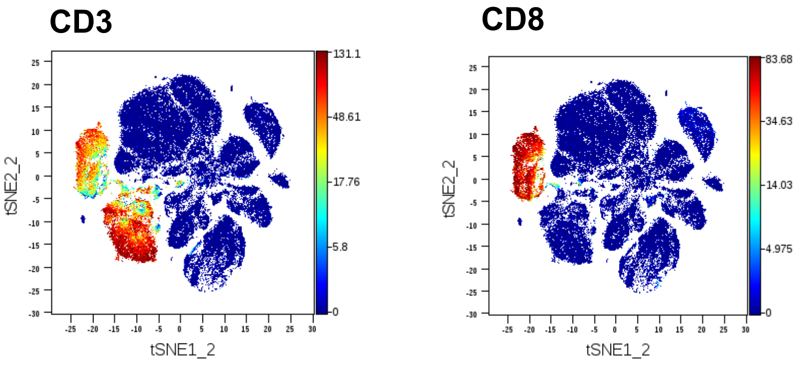

Cluster 8
B cell

Manual gating

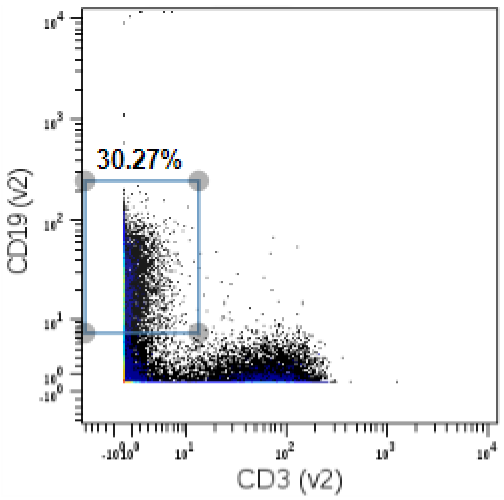

Overlay on viSNE

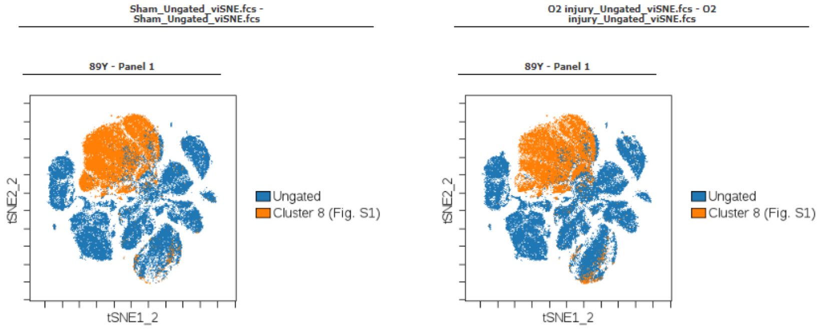

Expression of lineage markers

CD19

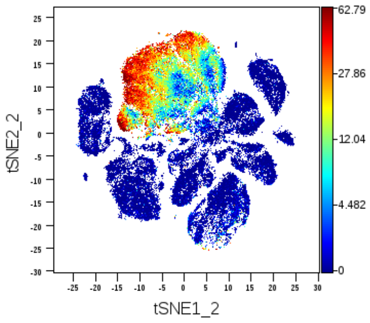

Cluster 9
NK cell

Manual gating

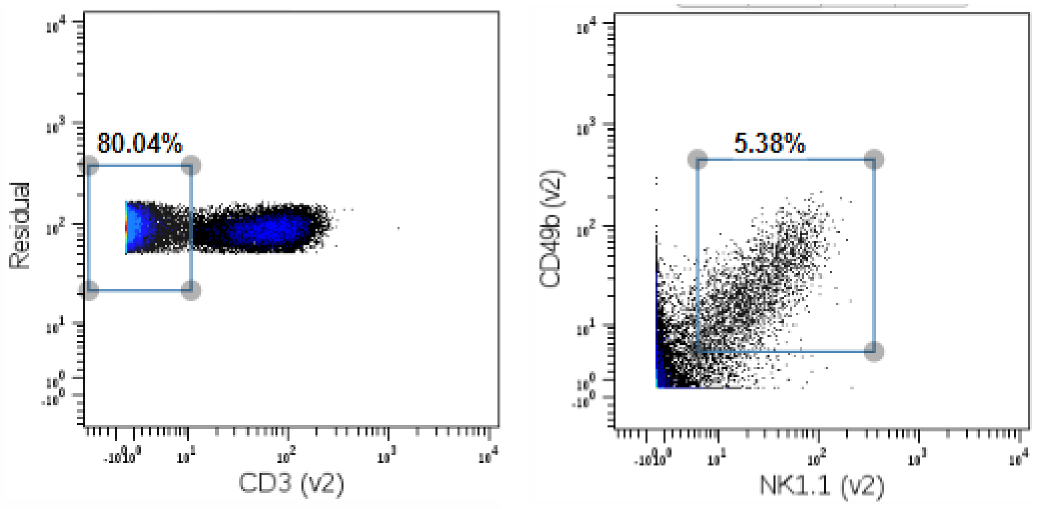

Overlay on viSNE

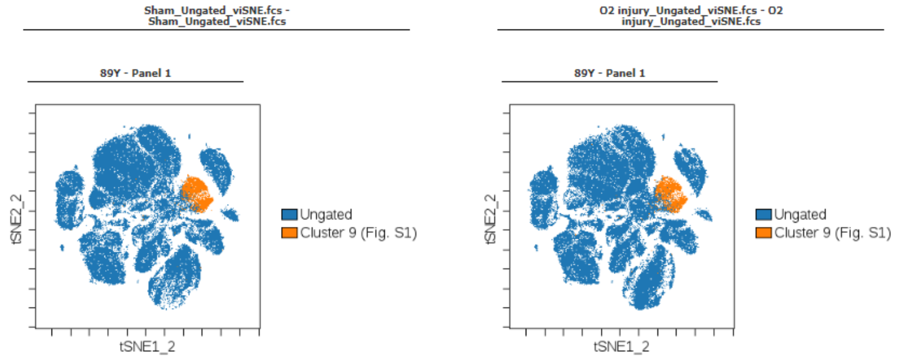

Expression of lineage markers

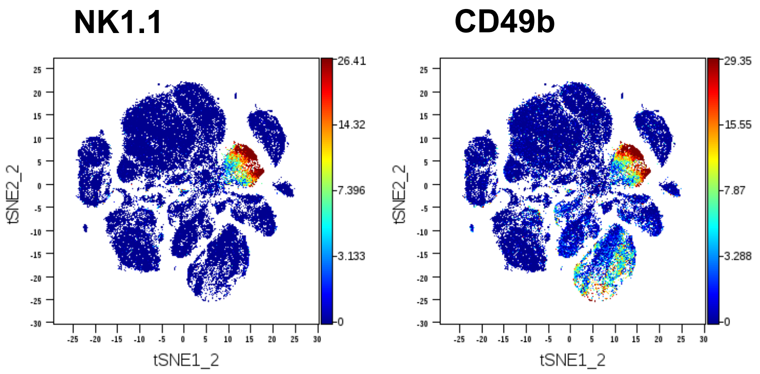

Figure S4

Cluster 1
Alveolar macrophage
CD68+ Siglec-F+
MHC low

Cluster 2
Alveolar macrophage
CD68+ Siglec-F+
MHC intermediate

Cluster 3
Myeloid cell
CD68+ Siglec-F-
MHC high

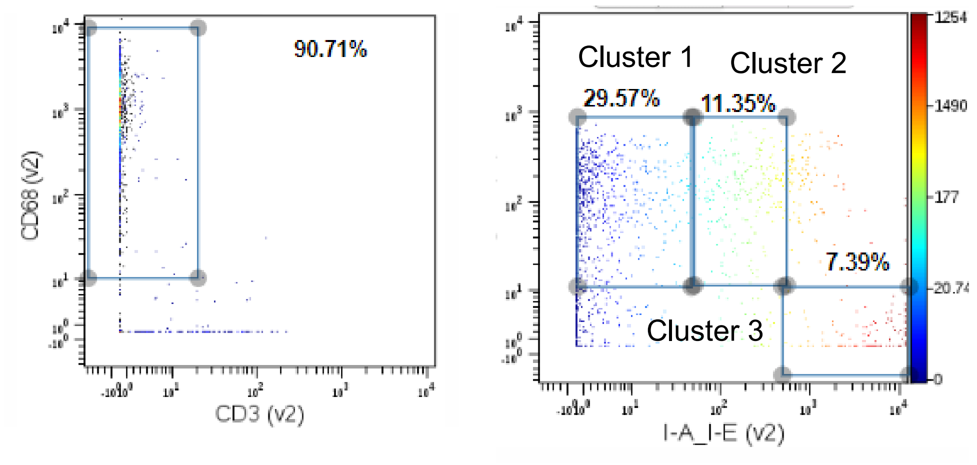

Overlay on viSNE

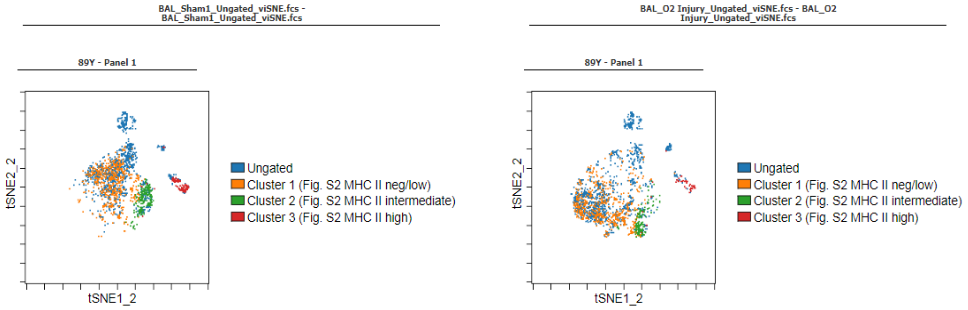

Expression of lineage markers

Siglec-F **CD68** **MHCII**

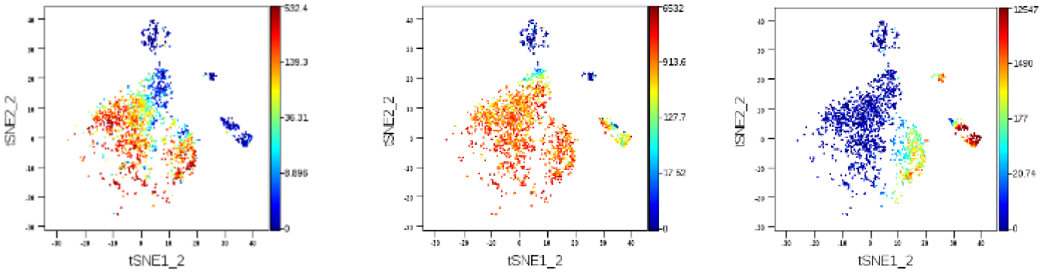

Manual gating

Cluster 4
B cell
CD19+

Cluster 5
T cell
CD3+

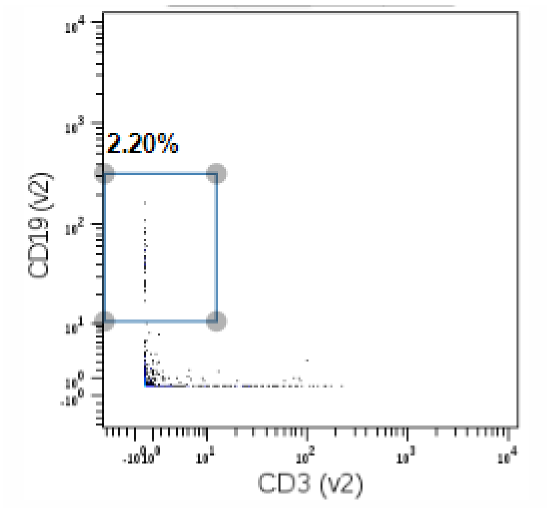

Overlay on viSNE

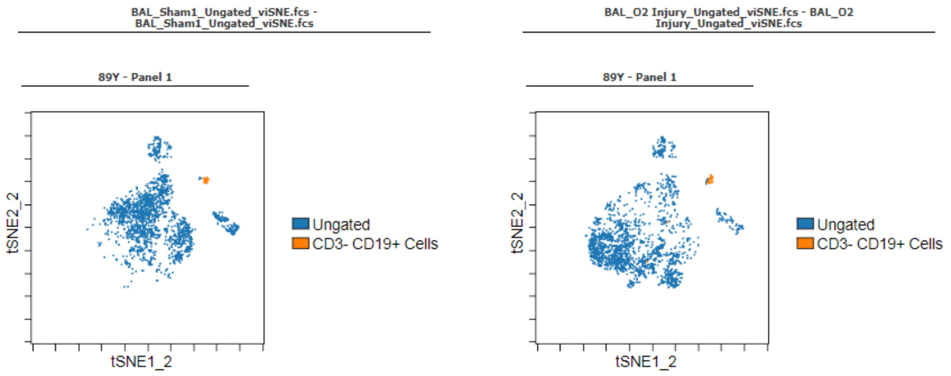

Expression of lineage markers

CD19 **CD3**

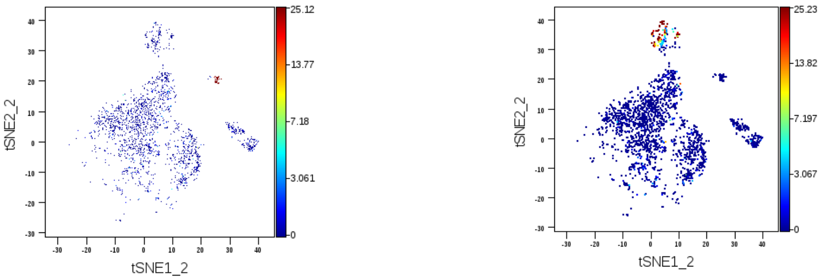

Figure S5

EXPERIMENT 1 (N=7)

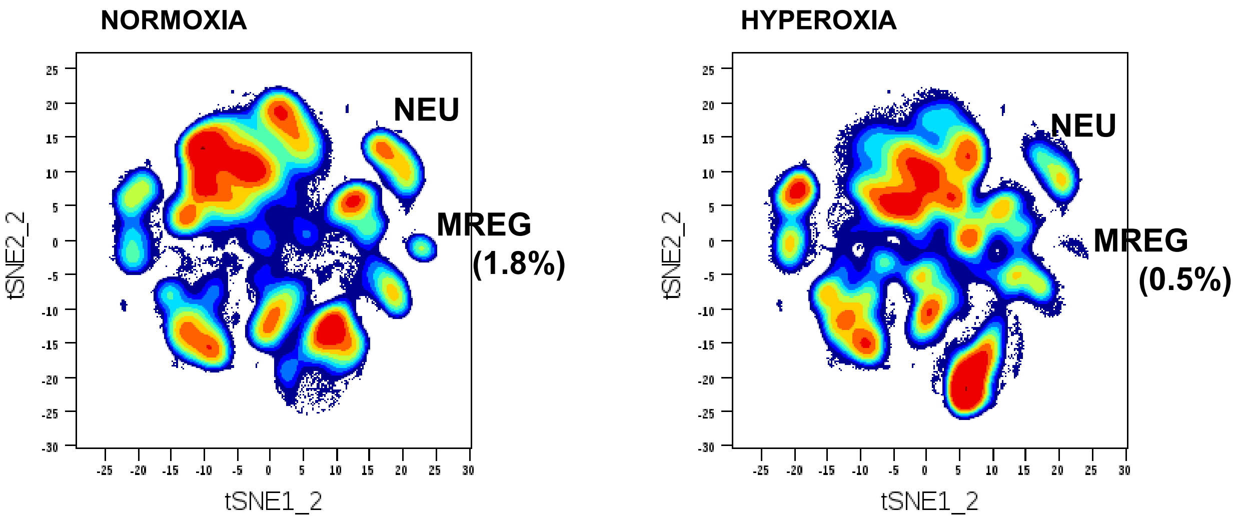

EXPERIMENT 2 (N=5)

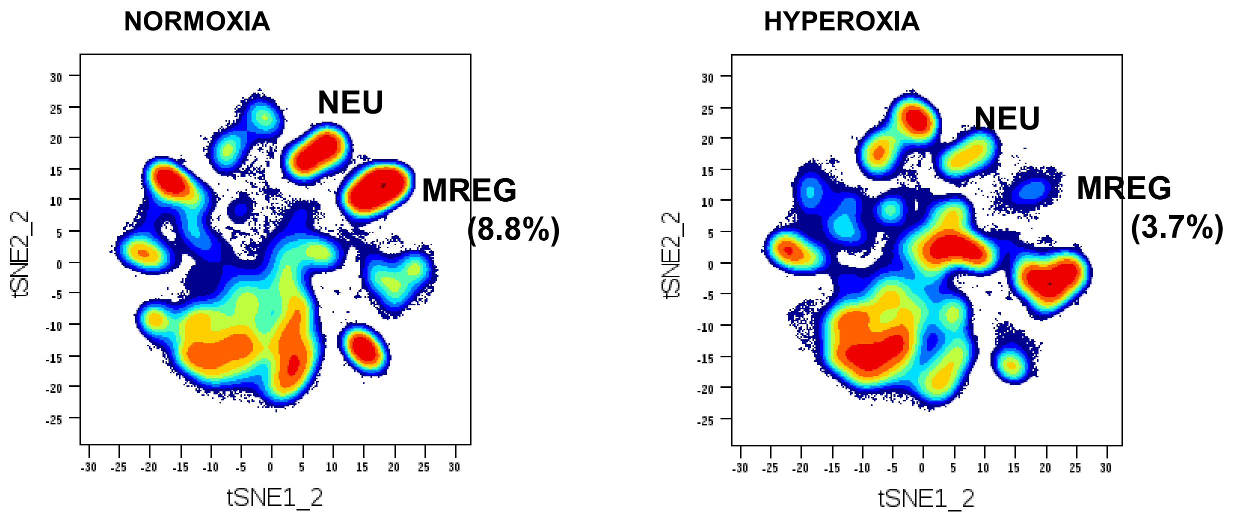

Figure S6

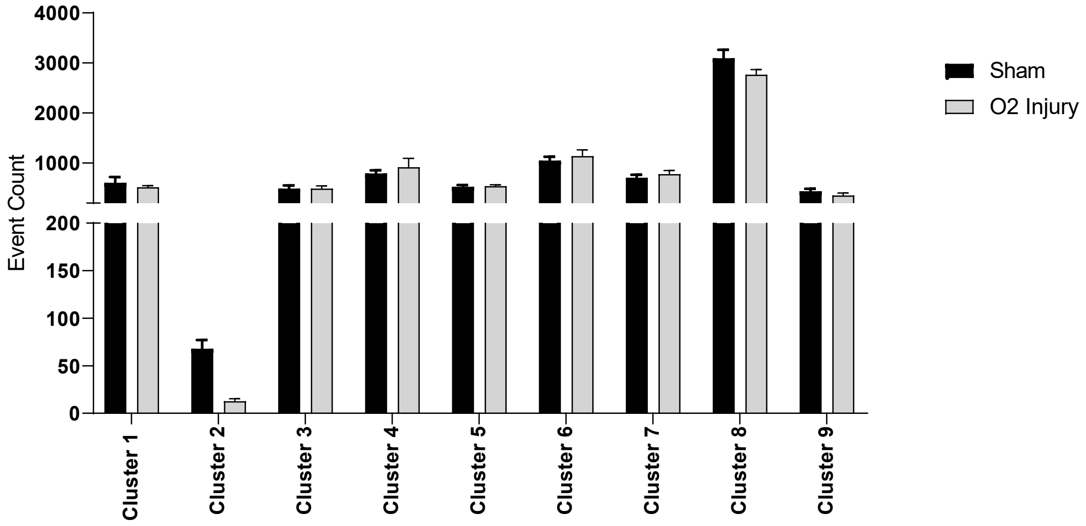

Cluster 1	neutrophils
Cluster 2	myeloid regulatory cells
Cluster 3	monocytes
Cluster 4	alveolar macrophages
Cluster 5	interstitial macrophages
Cluster 6	CD4+ T cells
Cluster 7	CD8+ T cells
Cluster 8	B cells
Cluster 9	NK cells

Figure S7

EXPERIMENT 1 (N=7)

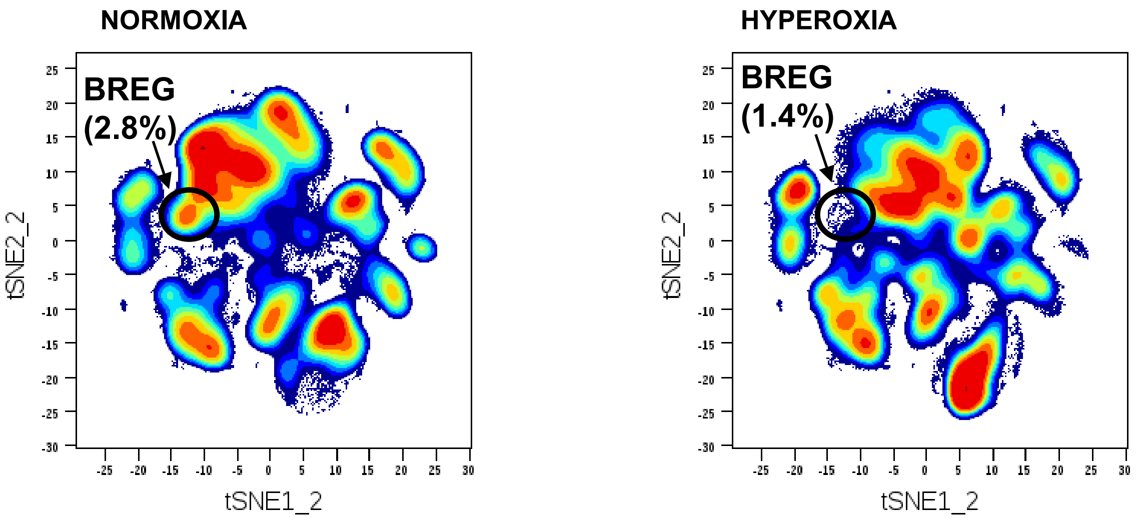

EXPERIMENT 2 (N=5)

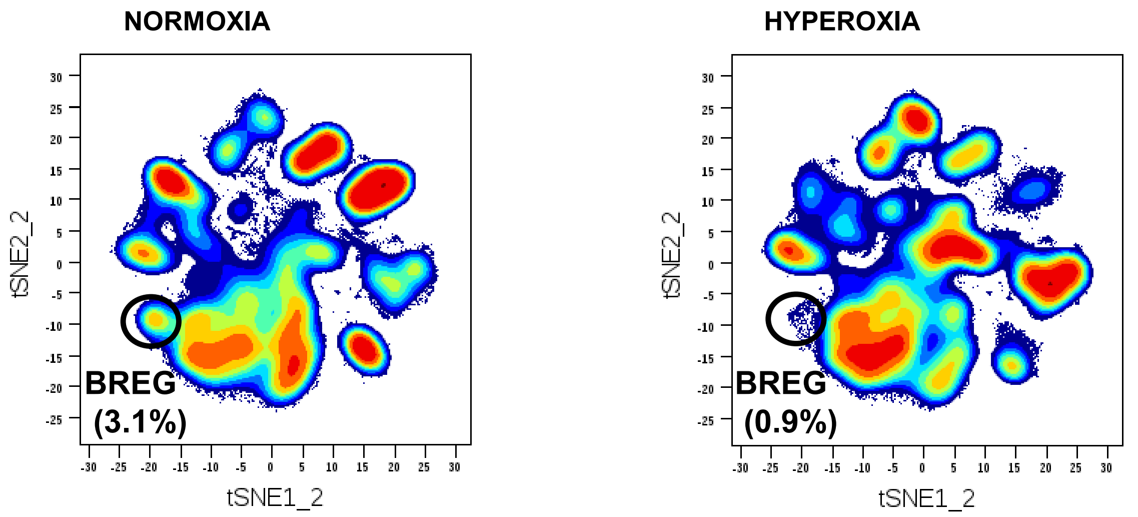

Figure S8

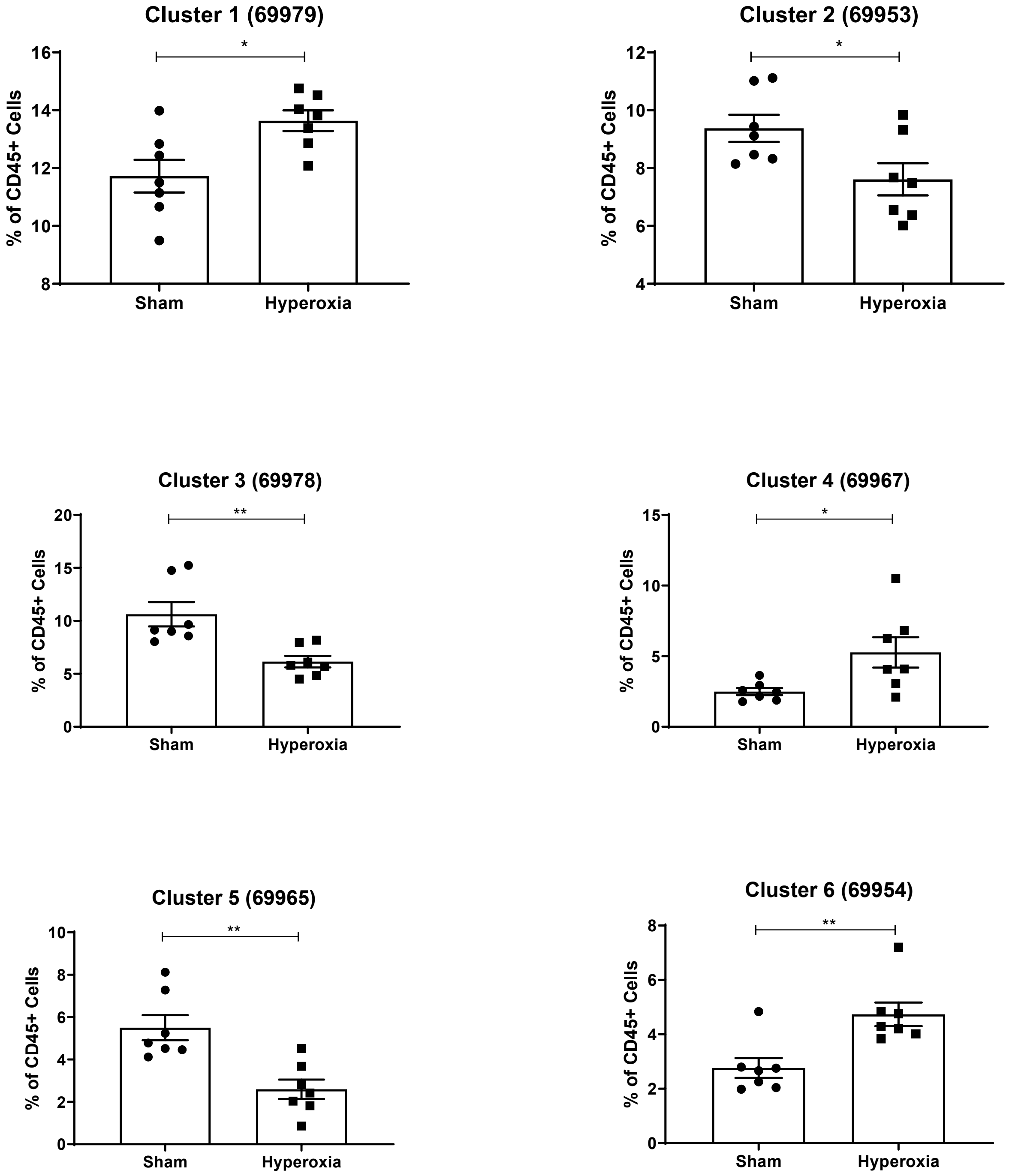

Cluster 1 (69979)
Cluster 2 (69953)
Cluster 3 (69978)
Cluster 4 (69967)
Cluster 5 (69965)
Cluster 6 (69954)
% of CD45+ Cells
Sham
Hyperoxia
*
**

Table S1

Marker	Clone	Metal
EpCAM	G8.8	113In
CD44	IM7	115In
CD45	30-F11	141Pr
CD8a	53-6.7	142Nd
CD73	TY/11.8	143Nd
VEGF R1	141522	144Nd
CD4	RM4-5	145Nd
CD11c	N418	146Nd
Ly6G	1A8	148Nd
PD-L1	10F.9G2	149Sm
CX3CR1	SA011F11	150Nd
Ly6C	HK1.4	151Eu
CD3	145-2C11	152Sm
CD172a	P84	153Eu
CD103	2E7	154Sm
CD68	FA-11	155Gd
CD19	6D5	156Gd
CD205	NLDC-145	158Gd
C39	Duha59	159Tb
Sca-1	E13-161.7	160Gd
Arginase I	Poly	161Dy
Foxp3	FJK-16s	162Dy
NK1.1	PK136	163Dy
Ki67	16A8	164Dy
CD115	460615	165Ho
CD86	GL-1	166Er
Grz B	GB11	167Er
CD11b	M1/70	169Tm
Siglec-F	E50-2440	170Er
CD49b	DX5	171Yb
TCRgd	GL3	172Yb
CD69	H1.2F3	173Yb
I-A/I-E	M5/114.15.2	174Yb
F4/80	BM8	175Lu
TLR2	T2.5	176Yb
Autotaxin	Polyclonal	209Bi

Figure S1. Lung tissue during normoxia and hyperoxia. Lung parenchyma from WT mouse during normoxia and after 48 hours of hyperoxia. Representative sample, H&E staining, 400x magnified. No evidence of diffuse alveolar damage is seen at 48 hours.

Figure S2. Stepwise gating approach to identify live CD45+ immune cells for CyTOF analysis. Step 1: Single cells were gated based on DNA content (193Ir-DNA2+). Step 2: Elimination of normalization beads. Step 3: Gating on live cells. Step 4: Gating on CD45+ cells.

Figure S3. Identification of immune cell lineage of cell clusters in the whole lung based on the expression of surface and intracellular markers. Overlays of manually gated populations on tSNE plots.

Figure S4. Identification of immune cell lineage of cell clusters in BALF based on the expression of surface and intracellular markers. Overlays of manually gated populations on tSNE plots.

Figure S5. Myeloid regulatory cells during normoxia and hyperoxia. tSNE plots from 2 independent experiments (N=7 and N=5) show depletion of myeloid regulatory cells during hyperoxia.

Figure S6. Absolute cell numbers of 9 major immune clusters as acquired by CyTOF in the live gate before equal sampling (7 control mice and 7 mice in hyperoxia).

Figure S7. Regulatory B cells during normoxia and hyperoxia. tSNE plots from 2 independent experiments (N=7 and N= 5) show depletion of regulatory B cells during hyperoxia.

Figure S8. Comparison of frequencies of CITRUS-identified clusters during normoxia and hyperoxia with unpaired t-test . Each dot represents data from one lung. * indicated $p < 0.05$, ** indicated $p < 0.01$.

Table S1. Panel of CyTOF antibodies used in the study. 36 metal conjugated antibodies were used in the study.
